# Prevalence and associated factors of premature discontinuation of antiplatelet therapy after ischemic stroke: a nationwide population-based study

**DOI:** 10.1186/s12883-021-02384-5

**Published:** 2021-09-10

**Authors:** Seung Jae Kim, Oh Deog Kwon, Ho Chun Choi, Eung-Joon Lee, BeLong Cho, Dae Hyun Yoon

**Affiliations:** 1 International Healthcare Center, Seoul St. Mary’s Hospital, College of Medicine, The Catholic University of Korea, Seoul, 06591 Republic of Korea; 2 Department of Family Medicine, Seoul St. Mary’s Hospital, College of Medicine, The Catholic University of Korea, Seoul, 06591 Republic of Korea; 3 Movinci Clinic, Seoul, 06030 Republic of Korea; 4 Department of Family Medicine, Healthcare System Gangnam Center, Seoul National University Hospital, Seoul, 06236 Republic of Korea; 5 Nuvizen, Palo Alto, California 94303 USA; 6 Institute of Public Health and Medical Care, Seoul National University Hospital, Seoul, 03080 Republic of Korea; 7 Department of Neurology, Seoul National University Hospital, Seoul, 03080 Republic of Korea; 8 Department of Family Medicine, Seoul National University Hospital, Seoul, 03080 Republic of Korea; 9 Department of Psychiatry, Healthcare System Gangnam Center, Seoul National University Hospital, Seoul, 06236 Republic of Korea

**Keywords:** Ischemic stroke, Antiplatelet, Premature discontinuation, Associated factors

## Abstract

**Background:**

We tried to evaluate the prevalence of premature discontinuation of antiplatelets and its affecting factors after ischemic stroke using large-sized representative national claims data.

**Methods:**

Patients aged 20 years or older with newly confirmed ischemic stroke who started aspirin or clopidogrel for the first time were selected from 2003 to 2010 National Health Insurance Service-National Sample Cohort (NHIS-NSC) of South Korea (*n* = 4621), a randomly collected sample which accounts for 2.2% (*n* = 1,017,468) of total population (*n* = 46,605,433). The prevalence of discontinuation of antiplatelets was measured every 6 months until the 24 months since the first prescription. Then we classified the participants into 2 groups according to the discontinuation status at 12 months and assessed the factors influencing premature discontinuation of antiplatelets within 12 months.

**Results:**

Among total participants, 35.5% (*n* = 1640) discontinued antiplatelets within 12 months and 58.5% (*n* = 2704) discontinued them within 24 months. The remaining 41.5% (*n* = 1917) continued them for 24 months or more. In the multivariate logistic regression analysis, initiating treatment with aspirin monotherapy [adjusted OR (aOR), 2.66, 95% CI 2.17–3.25] was the most prominent determinant of premature discontinuation within 12 months followed by CCI score ≥ 6 (aOR 1.50, 95% CI 1.31–1.98), and beginning treatment with clopidogrel monotherapy (aOR 1.41, 95% CI 1.15–1.72). Rural residency (aOR 1.36, 95% CI 1.14–1.62), < 4 total prescribed drugs (aOR 1.24, 95% CI 1.05–1.47), lower income (aOR 1.20, 95% CI 1.03–1.40 for middle income class and OR 1.21, 95% CI 1.02–1.45 for low income class), and ages ≥70 years (aOR 1.15, 95% CI 1.00–1.31) were also significantly associated with premature discontinuation of antiplatelets within 12 months.

**Conclusions:**

The prevalence of premature discontinuation of antiplatelets after ischemic stroke was quite high. Thus, by understanding factors associated with premature discontinuation, a more strategic approach is required for the physicians to improve persistence with antiplatelets.

## Introduction

Stroke is one of the leading causes of mortality and disability worldwide, and its burden of disease has been continuously increasing [1, 2]. Among all the cases of strokes, 87% are ischemic [3]. Patients with this kind of stroke have relatively high risks for recurrent stroke, myocardial infarction (MI), and mortality throughout the years [4, 5].

Antiplatelet therapy (APT), with agents such as aspirin and clopidogrel, is the cornerstone of secondary prevention of ischemic stroke, as it has been proven to reduce its recurrence and prevent other cardiovascular complications, including mortality [6–8]. Despite this, non-adherence with secondary preventive antiplatelet agents has been widespread among patients who suffered from cardiovascular diseases (CVD) as this was a main risk factor for treatment failures and poor clinical outcomes [9]. Moreover, previous studies have reported that the majority of discontinuations of antiplatelet agents occurred within 1–2 years since the initial ischemic stroke [10–14], and these discontinuations were directly associated with higher incidence of stroke recurrence, MI, and vascular mortality [14–16]. In addition, the causes of medication non-adherence are generally multifactorial [17]. The World Health Organization (WHO) has classified potential factors that could cause poor medication adherence into 5 interacting dimensions, which include patient, condition, therapy, socioeconomic, and health system-related factors [17, 18]. Although there have been some studies that investigated the factors affecting the early discontinuation of APT after ischemic stroke [10, 12, 13, 16], relatively few studies have been conducted with a large-sized real-world data. Thus, using the large-sized nationally representative claim database of South Korea, the purpose of this study was to examine the prevalence of and identify the factors associated with the premature discontinuation of APT in patients with newly confirmed ischemic stroke.

## Methods

### Source of data

The data of our study were derived from the National Health Insurance Service-National Sample Cohort (NHIS-NSC). NHIS-NSC is a population-based claims database established in 2002 by the National Health Insurance Service (NHIS) of South Korea. The NHIS offers universal health insurance coverage to all individuals with Korean citizenship through mandatory enrollment. NHIS-NSC consists of 1,017,468 citizens, which accounts for 2.2% of the total eligible Korean population of 46,605,433. The NHIS-NSC is collected every year via continuous investigation, and it includes qualification data, medical service claims, and pharmacy claims data. Qualification data contain patients’ basic information, such as age, sex, household income, insurance type, region of residence, mortality information, and others. On the other hand, medical service claims data contain all insurance-covered medical records, including diagnosis records, inpatient and outpatient healthcare service records, billing statements, etc. Meanwhile, pharmacy claims data include information such as generic names of prescribed drugs, dates of prescription, total supplied days of drugs per outpatient visit, medication dosages and frequencies, and others [19]. The detailed information regarding the representativeness and validity of NHIS-NSC have been described in other studies [19, 20].

### Study population

**Fig. 1:**
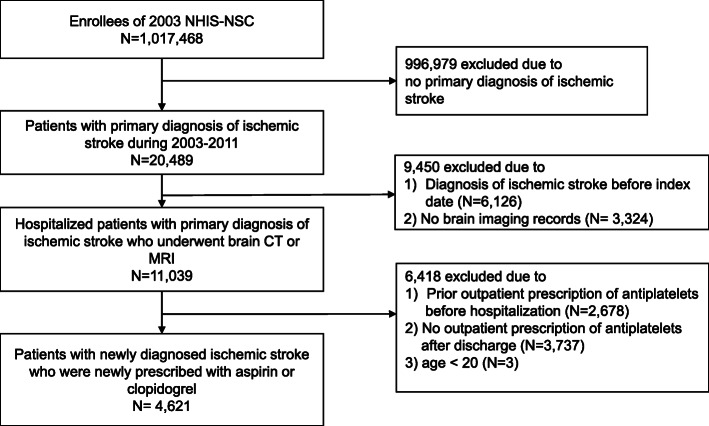
Flow diagram of selection of study population. NHIS-NSC, National Health Insurance Service-National Sample Cohort; CT, computed tomography; MRI, Magnetic resonance imaging

Participants newly confirmed with ischemic stroke aged ≥20 years old, who were newly started with APT from years 2003 to 2010, were selected from the NHIS-NSC (*n* = 20,489). The definition of ischemic stroke was hospitalization with a primary diagnosis of ischemic stroke (International Classification of Disease, 10th revision, ICD-10: I63, I64, I65, I66) with a record of brain computed tomography (CT) or magnetic resonance imaging (MRI) during hospitalization (*n* = 11,039). We only included patients with brain imaging records because we assumed that patients with ischemic stroke would undergo such brain imaging studies [21]. This definition was also adapted by other studies involving post-stroke patients that used data from the NHIS-NSC [22, 23]. Participants with diagnostic histories of ischemic stroke (I63-I66) or outpatient prescription histories of antiplatelet agents prior to the index hospitalization dates were excluded. As such, we only included patients with new ischemic stroke diagnosis who initiated antiplatelet agents for the first time. We limited antiplatelet agents to aspirin or clopidogrel, the most commonly used antiplatelets for the secondary prevention of ischemic stroke [7, 8, 24], according to the Anatomical Therapeutic Chemical (ATC) classification code [25]. Other antiplatelet agents besides aspirin or clopidogrel were excluded since these were rarely prescribed because these were not covered by Korean health insurance during the study period. Participants without outpatient prescription records of aspirin or clopidogrel after discharge were also excluded. As a result, the final study population consisted of 4621 participants (Fig. 1).

### Outcome variables

Each participants’ outpatient prescriptions of antiplatelets were observed for at least 2 years, starting from the date of the first outpatient prescription. We first measured the prevalence of discontinuation of antiplatelets at 6, 12, 18, and 24 months. Thereafter, we divided the participants into 2 groups according to the discontinuation status at 12 months and evaluated the factors associated with premature discontinuation of antiplatelets within 12 months. Discontinuation was defined as when the antiplatelet agents were discontinued without refills throughout the rest of the observation period. Participants were viewed as continuers as long as they were prescribed with either aspirin or clopidogrel throughout observation period regardless of replacing one drug to another. For patients on dual antiplatelet therapy (DAPT) of aspirin and clopidogrel, it was defined as discontinuation only when both agents were discontinued without refills throughout the remainder of the observation period.

### Covariates

According to the WHO, adherence to medication is affected by the interactions of patient, condition, therapy, socioeconomic, and health system-related factors [17, 18]. For our study, the patient-related factors included participants’ age and sex; the Charlson comorbidity index (CCI) for the condition-related factors; type of initiated antiplatelet agents and number of prescribed drugs for the therapy-related factors; the socioeconomic-related factors consisted of household incomes and residential areas; and lastly, the type of insurance for the health system-related factors. These variables were derived as covariates from NHIS-NSC and were all adjusted in the analysis. The CCI score was measured at the beginning of the observation period, based on the ICD-10 codes [26]. The number of prescribed drugs was defined as the average total number of prescribed medications during the observation period. Urban residency was defined as those who resided in metropolitan cities or cities while those who were not urban dwellers were considered as rural dwellers. Household income was converted into 3 income tertiles on a scale of 0 (poorest) to 10 (wealthiest).

### Statistical analysis

Descriptive analyses were performed to compare the characteristics of patients who discontinued APT within 12 months and those who continued APT for 12 months or more. Mean ± standard deviation (SD) was used to demonstrate continuous variables, while percentage was used to summarize categorical variables. The overall tendency of characteristics between each group were compared by chi-square test. To investigate the factors associated with the premature discontinuation of antiplatelets, unadjusted univariate and adjusted multivariate logistic regression analyses were performed between the two study groups. Factors that demonstrated *p*-values of less than 0.1 in univariate analyses were included in the multivariate analysis to test their independence. In these analyses, patients who passed away within 12 months were excluded. All statistical analyses were conducted with STATA version 14.1 (Stata Corp., College Station, TX, USA) and statistical significance was defined as a p-value of less than 0.05.

## Results

### Baseline characteristics of total participants

Of the 4621 total participants, 56.2% were male and 43.8% were female. The mean age of the total participants was 66.4 ± 12.3 years, with 70.9% of them aged ≥60 years. Majority of the participants (84.8%) were residing in urban areas and the upper-middle income class accounted for 72.9%. Almost all of the participants (92.8%) were benefiting from Medicare, while the remaining 7.2% were Medical Aid beneficiaries. In terms of comorbidities, 47.9% of the patients had CCI scores of less than 3, 46.5% had CCI scores between 3 to 5, while 5.6% had CCI scores 6 or higher. Regarding the type of antiplatelet agents, 36.5% of participants initiated monotherapy with aspirin, 47.0% with clopidogrel, and 16.5% initiated with DAPT. Regarding the polypharmacy status, 16.7% of patients were taking less than 4 total prescribed drugs, 63.9% were taking 4 to 7 total drugs, and 19.4% were taking at least a total of 8 prescribed medications. The average duration of APT of all the participants was 541.1 ± 354.5 days (Table 1).

**Table 1:** Baseline Characteristics of total participants

Characteristics	All n (%) or mean ± SD
Total	4621 (100%)
Sex	
Male	2550 (56.2%)
Female	2071 (43.8%)
Age (years)	
Mean	66.4 ± 12.3
20–49	481 (10.4%)
50–59	864 (18.7%)
60–69	1267 (27.4%)
70–79	1437 (31.1%)
≥ 80	572 (12.4%)
Income	
High	1901 (41.1%)
Middle	1468 (31.8%)
Low	1252 (27.1%)
Residential area	
Urban	3917 (84.8%)
Rural	704 (15.2%)
Health insurance	
Medicare	4287 (92.8%)
Medical aid	334 (7.2%)
Charlson comorbidity index	
< 3	2215 (47.9%)
3–5	2149 (46.5%)
≥ 6	257 (5.6%)
Initiated antiplatelets	
Aspirin	1688 (36.5%)
Clopidogrel	2170 (47.0%)
Dual therapy	763 (16.5%)
Number of prescribed medications	
< 4	771 (16.7)
4–7	2953 (63.9)
≥ 8	897 (19.4)
Average duration of antiplatelets (days)	541.1 ± 354.5

*SD* Standard deviation

### Prevalence of premature discontinuation of antiplatelets over time

**Fig. 2:**
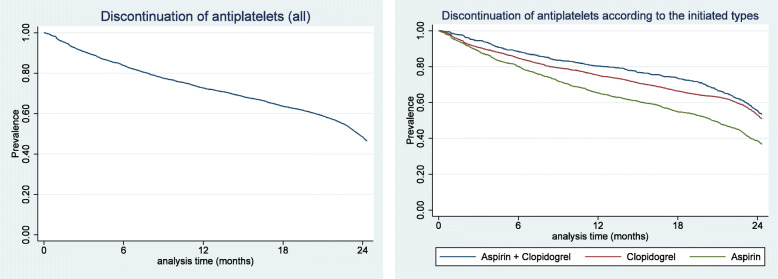
Prevalence of premature discontinuation of antiplatelets over time for ischemic stroke survivors

Among the 4621 newly confirmed ischemic stroke patients who started antiplatelets for the first time, 25.3% (*n* = 1170) prematurely discontinued intake within 6 months. In 103 of the 1170 cases, discontinuation was due to the patient’s death. An additional 470 (10.2%) premature discontinuations, 114 of which were caused by death, occurred over the next 6 months. Taken together, the prevalence of premature discontinuation of antiplatelets within 12 months was 35.5% (*n* = 1640). Between 12 and 18 months, 369 patients (97 because of death) discontinued antiplatelets, and 695 more (85 because of death) discontinued them between 18 and 24 months. Hence, the prevalence of early discontinuation within 24 months reached 58.5% (*n* = 2704) of total patients. The remaining 41.5% (*n* = 1917) continued antiplatelet intake for 24 months or more. In every 6 months of observation until the first 18 months since the first prescription of antiplatelet, those who initiated monotherapy with aspirin demonstrated the highest prevalence of premature discontinuation, followed by those who started monotherapy with clopidogrel. The prevalence of premature discontinuation was lowest among those who initiated treatment with DAPT. However, from 18 to 24 months since the first prescription of antiplatelets, patients who initiated treatment with DAPT displayed the highest prevalence of discontinuation, followed by those who began monotherapy with aspirin. The patients who started monotherapy with clopidogrel had the lowest prevalence of premature discontinuation at 18–24 months. Among the 1917 patients who continued antiplatelets for 24 months or more, those who began treatment with DAPT accounted for the highest proportion, followed by those who initiated with clopidogrel monotherapy, while the percentage of those who began with aspirin monotherapy was the lowest (Table 2., Fig. 2.).

**Table 2:** Prevalence of premature discontinuation of antiplatelets after ischemic stroke (n = 4621)

Variables	Discontinued < 6 months		Discontinued 6–12 months		Discontinued 12–18 months		Discontinued 18–24 months		Continued ≥24 months
	All	Discontinued due to death	All	Discontinued due to death	All	Discontinued due to death	All	Discontinued due to death	
	Prevalence n (%)	Prevalence n (%)	Prevalence n (%)	Prevalence n (%)	Prevalence n (%)	Prevalence n (%)	Prevalence n (%)	Prevalence n (%)	Prevalence n (%)
All antiplatelets (*n* = 4621)	1170 (25.3%)	103 (2.2%)	470 (10.2%)	114 (2.4%)	369 (8.0%)	97 (2.1%)	695 (15.0%)	85 (1.8%)	1917 (41.5%)
Aspirin (*n* = 1688)	545 (32.3%)	31 (1.8%)	215 (12.7%)	42 (2.5%)	148 (8.8%)	45 (2.7%)	253 (15.0%)	29 (1.7%)	527 (31.2%)
Clopidogerl (*n* = 2170)	491 (22.6%)	55 (2.5%)	197 (9.1%)	54 (2.5%)	171 (7.9%)	38 (1.8%)	302 (13.9%)	44 (2.0%)	1009 (46.5%)
Dual therapy (*n* = 763)	134 (17.6%)	17 (2.2%)	58 (7.6%)	18 (2.4%)	50 (6.6%)	14 (1.8%)	140 (18.3%)	12 (1.6%)	381 (49.9%)

### Characteristics comparison between antiplatelet continuers (≥ 12 months) and discontinuers (< 12 months)

The characteristics of the participants who discontinued antiplatelet agents within 12 months (*n* = 1423) and those who continued intake for 12 months or more (*n* = 2981) are presented in Table 3. In this comparison, participants who passed away within 12 months (*n* = 217) were excluded. The discontinuers took antiplatelets for 115.7 ± 116.0 days in average before discontinuing intake, while the average duration for those who continued for 12 months or more was 778.4 ± 170.4 days. Patients who prematurely discontinued antiplatelets within 12 months were more likely to be 70 years or older (44.1% vs. 40.4%), belonged to lower income classes (32.7% vs. 31.5% for middle income class and 29.2% vs 25.8% for low income class), were Medical aid beneficiaries (8.4% vs. 6.5%), resided in rural areas (18.4% vs. 13.5%), were initiated with monotherapy (88.9% vs. 80.8%), on aspirin when started with monotherapy (48.2% vs. 31.1%), had CCI scores ≥6 (6.3% vs. 4.6%), and had less than 4 total prescribed drugs (18.7% vs. 15.5%). The proportion of female was also slightly higher (46.6% vs. 43.8%), but this trend was not statistically significant.

**Table 3:** Baseline Characteristics according to the premature discontinuation at 12 months

Characteristics	Discontinued < 12 months^a^ n (%) or mean ± SD	Continued 12 ≥ months n(%) or mean ± SD	*p* value
Total	1423 (100%)	2981 (100%)	
Sex			0.086
Male	760 (53.4%)	1674 (56.2%)	
Female	663 (46.6%)	1307 (43.8%)	
Age (year)			0.022
Mean	66.6 ± 12.6	65.5 ± 12.0	
< 70	796 (55.9%)	1776 (59.6%)	
≥ 70	627 (44.1%)	1205 (40.4%)	
Income			0.008
High	542 (38.1%)	1274 (42.7%)	
Middle	465 (32.7%)	939 (31.5%)	
Low	416 (29.2%)	768 (25.8%)	
Residential area			0.000
Urban	1161 (81.6%)	2578 (86.5%)	
Rural	262 (18.4%)	403 (13.5%)	
Health insurance			0.023
Medicare	1303 (91.6%)	2786 (93.5%)	
Medical aid	120 (8.4%)	195 (6.5%)	
Charlson comorbidity index			0.017
< 6	1334 (93.7%)	2845 (95.4%)	
≥ 6	89 (6.3%)	136 (4.6%)	
Initiated antiplatelets			0.000
Dual therapy	158 (11.1%)	571 (19.2%)	
Clopidogrel	579 (40.7%)	1482 (49.7%)	
Aspirin	686 (48.2%)	928 (31.1%)	
Number of prescribed medications			0.008
< 4	266 (18.7%)	463 (15.5%)	
≥ 4	1157 (81.3%)	2518 (84.5%)	
Average duration of antiplatelets (days)	115.7 ± 116.0	778.4 ± 170.4	

*SD* Standard deviation

^a^ Excluded participants (*n* = 217) who died within 12 months

### Factors associated with premature discontinuation of antiplatelets within 12 months

The univariate and multivariate logistic regression analyses between the patients who discontinued antiplatelet within 12 months and those who continued intake for 12 months or more are shown in Table 4. In the univariate analysis, initiating monotherapy with aspirin was the most prominently correlated factor with premature discontinuation of antiplatelets within 12 months [Odds ratio (OR) 2.67, 95% confidence interval (CI) 2.18–3.27], followed by rural residency (OR 1.44, 95% CI 1.22–1.71), and initiating monotherapy with clopidogrel (OR 1.41, 95% CI 1.16–1.73 for clopidogrel). The following factors were also significantly associated with the premature discontinuation of antiplatelet agents: CCI scores ≥6 (OR 1.40, CI 1.06–1.84), being a Medical Aid beneficiary (OR 1.32, 95% CI 1.04–1.67, having less than 4 total prescribed drugs (OR 1.25, 95% CI 1.06–1.48), lower income (OR 1.16, 95% CI 1.00–1.35 for middle income class and OR 1.27, 95% CI 1.09–1.49 for low income class), and ages of 70 years or older (OR 1.16, 95% CI 1.02–1.32). The results of the multivariate analysis were substantially similar to those of the univariate analysis. Starting monotherapy with aspirin [adjusted OR (aOR), 2.66, 95% CI 2.17–3.25 for aspirin] remained to be the most influential factor for premature discontinuation of antiplatelets within 12 months, followed by CCI score ≥ 6 (aOR 1.50, 95% CI 1.13–1.98) and initiating monotherapy with clopidogrel (aOR 1.41, 95% CI 1.15–1.72). Furthermore, similar to the results of the univariate study, the following factors were also significantly associated with premature discontinuation: rural residency (aOR 1.36, 95% CI 1.14–1.62), less than 4 total prescribed drugs (aOR 1.24, 95% CI 1.05–1.47), lower income (aOR 1.20, 95% CI 1.03–1.40 for middle income class and OR 1.21, 95% CI 1.02–1.45 for low income class), and ages of 70 years or older (aOR 1.15, 95% CI 1.00–1.31). However, the correlation of being a Medical Aid beneficiary was no longer statistically significant in the multivariate analysis.

**Table 4:** Factors associated with premature discontinuation of antiplatelets within 12 months compared to continuers

Factors	Univariate logistic regression analysis		Multivariate logistic regression analysis	
	Crude OR (95% CI)	*p* value	Adjusted OR^a^(95% CI)	*p* value
Total				
Sex				
Male	1 (reference)		1 (reference)	
Female	1.12 (0.98–1.27)	0.086	1.03 (0.90–1.17)	0.688
Age				
< 70	1 (reference)		1 (reference)	
≥ 70	1.16 (1.02–1.32)	0.022	1.15 (1.00–1.31)	0.044
Income				
High	1 (reference)		1 (reference)	
Middle	1.16 (1.00–1.35)	0.047	1.20 (1.03–1.40)	0.021
Low	1.27 (1.09–1.49)	0.002	1.21 (1.02–1.45)	0.033
Residential area				
Urban	1 (reference)		1 (reference)	
Rural	1.44 (1.22–1.71)	0.000	1.36 (1.14–1.62)	0.001
Health insurance				
Medicare	1 (reference)		1 (reference)	
Medical aid	1.32 (1.04–1.67)	0.023	1.18 (0.89–1.55)	0.204
Charlson comorbidity index				
< 6	1 (reference)		1 (reference)	
≥ 6	1.40 (1.06–1.84)	0.018	1.50 (1.13–1.98)	0.005
Initiated antiplatelets				
Dual therapy	1 (reference)		1 (reference)	
Clopidogrel	1.41 (1.16–1.73)	0.001	1.41 (1.15–1.72)	0.001
Aspirin	2.67 (2.18–3.27)	0.000	2.66 (2.17–3.25)	0.000
Number of prescribed medications				
≥ 4	1 (reference)		1 (reference)	
< 4	1.25 (1.06–1.48)	0.008	1.24 (1.05–1.47)	0.013

*OR* Odds ratio

^a^ Adjusted for sex, age, income, residential area, type of health insurance, Charlson comorbidity index, type of initiated antiplatelets, and number of prescribed medications

## Discussion

In this real-world population-based cohort study, the prevalence of premature discontinuation with antiplatelet drugs was quite high for ischemic stroke survivors. More than one-third (35.4%) of the total participants prematurely discontinued APT during the first 12 months since the initiation of therapy, while more than half (58.3%) of patients discontinued them within 24 months. These statistics were found to be still significant, even after excluding those who discontinued antiplatelets due to death (as the prevalence of discontinuation at 12 and 24 months excluding deceased patients were 30.8 and 49.8%, respectively). These findings were consistent with the reports of other studies that nearly half of the participants discontinued APT within the first 24 months after ischemic stroke [11, 16, 27]. Thus, it is safe to claim that we have reconfirmed that non-persistence with APT among patients with ischemic stroke is a major problem and that the importance of maintaining antiplatelet intake cannot be emphasized enough.

Identifying the specific predictors of early discontinuation of APT is critical in planning proper strategies to maximize medication adherence among ischemic stroke survivors. In the present study, the results of the multivariate logistic regression analysis revealed that age (70 years or older), lower income, rural residency, more co-morbidities (CCI score ≥ 6), the type of initiated antiplatelet agents, and having less than 4 total prescribed medications were independent factors affecting the premature discontinuation of antiplatelets within the first 12 months.

Interestingly, our findings demonstrated that initiating with antiplatelet monotherapy was strongly associated with early discontinuation compared to DAPT. This result is in contrast with the general belief that medication adherence will be lower when a patient is taking two different drugs at the same time, compared to taking just one. We assume that this finding could be interpreted by applying the Health Belief Model [28, 29]. Patients who believe they are sick are more likely to possess healthy behaviors, including being more adherent to prescribed medications to maintain their well-being [28, 29]. DAPT is usually initiated among those with higher risk of recurrent vascular events, such as large artery atherosclerosis or the coexistence of coronary artery disease [30, 31]. Thus, there is a greater possibility that patients who initiated treatment with DAPT had more severe vascular diseases and this may have led them to be more persistent with their prescribed medications compared to those who began treatment with monotherapy.

In addition, initiating treatment with aspirin monotherapy after ischemic stroke was the most prominent predictor of early discontinuation of antiplatelet agents in our study. Even though APT is a fundamental part of secondary prevention of ischemic stroke patients, it is also associated with substantial risks of bleeding [gastrointestinal (GI) and intracranial] and symptoms related to gastrointestinal toxicity [32]. These were one of the leading causes for discontinuation of antiplatelets among Asian patients who received drug-eluting stent implantation for MI [33]. Furthermore, a previous randomized control trial has proved that clopidogrel has less risks of developing the adverse effects of both bleeding complications or GI symptoms compared to aspirin [34]. Therefore, we could assume that patients who initiated treatment with clopidogrel monotherapy could have been more persistent due to having less overall side effects compared to aspirin monotherapy initiators.

Another notable finding of this study was that participants with less than 4 total prescribed drugs presented with higher risks of premature discontinuation of antiplatelets compared to patients with 4 or more total prescribed drugs. On the contrary, previous studies reported that fewer total prescribed medications were associated with better persistence with antiplatelets after ischemic stroke [10, 12, 14].

The Health Belief Model could be applied to this finding as well since the overall health status of participants with fewer total prescribed medications are likely to be better than those on polypharmacy. Therefore, the importance of taking drugs as prescribed could have been overlooked for patients with 1–3 total prescribed drugs since they might have felt that their medical conditions were less serious than those of the patients with 4 or more total prescribed drugs. This trend was also found in other studies which investigated the medication adherence of patients with chronic diseases, such as hypertension and dyslipidemia. The Health Belief Model was also applied for the explanation of those findings [35, 36].

However, it seemed that even the explanation provided by the Health Belief Model could not facilitate good adherence to antiplatelets when patients’ general medical conditions were too severe as having more comorbidities (CCI score of 6 or higher) significantly affected the early discontinuation of antiplatelets after ischemic stroke. The presence of more comorbidities is a well-known predictor of poor functional outcomes for ischemic stroke survivors, including limitations of daily activities [37–40]. Thus, it is likely that patients with CCI scores of 6 or higher could not continuously take antiplatelets due to poorer functional ability, compared to those with CCI scores less than 6. This trend was also consistent with a previous study where it was found that an increase in comorbidities was a predictor for lower antihypertensive adherence [41].

Older age was also an influential factor of premature discontinuation of antiplatelets in this study, as discontinuation was significant among elderly patients who were 70 years or older. A previous study also noted that elderly ischemic stroke survivors were more vulnerable to poor medication adherence [14]. The prevalence of both cognitive and physical function impairments undeniably increases with age. Other studies have confirmed that these conditions are risk factors for poor medication adherence in elderly patients [42–44]. We also believe that the occurrence of physical and cognitive impairments was the reason for the positive association between elderly age and non-persistent with antiplatelets in our study.

Lastly, socioeconomic-related factors of lower income and rural residency independently predicted the non-persistence with APT. In previous studies conducted with acute MI patients who received drug-eluting stent placement, lower income was a predictor [45], while cost was one of the main reasons for premature discontinuation of APT [46]. Our result with ischemic stroke patients could also be understood on this basis.

In the case of rural residency, rural residents are generally confronted with various barriers when accessing to health care services, such as limited health care institutions, lack of physicians, less opportunity to consult specialists, financial constraints, social isolation, transportation difficulties, and longer distances to healthcare facilities [47, 48]. Our result suggest that these barriers may have contributed to the non-persistence with APT for ischemic stroke survivors residing in rural areas. A previous study conducted in Korea has also identified rural residency as a risk factor for non-adherence to antihypertensive medications [49].

The strength of the present study is that by analyzing representative national claims data consisting large populations, we objectively evaluated the persistence with antiplatelet intake of patients with ischemic stroke in a real-world setting. Furthermore, we considered various factors including age, sex, income, residential area, comorbidities, number of total prescribed drugs, type of health insurance, and initiated antiplatelets that could affect medication persistence in the analysis. Among these, we identified the significant predictors for premature discontinuation of antiplatelets.

Meanwhile, this study has several limitations as well. First, other important relevant factors that could affect the medication persistence, such as severity of the stroke, disability after stroke, disposition after discharge, caregiver status, and educational status could not be identified due to the nature of the NIHS-NSC database. Second, we were also unable to examine specific causes for discontinuation of antiplatelets, including adverse effects such as bleeding and GI toxicity. Thus, further research evaluating the reasons for non-persistence with APT for ischemic stroke survivors is needed. Third, prescription status of anticoagulation therapy (ACT) was not assessed in this study. Some ischemic strokes are consequence of atrial fibrillation, which is not always detected during the hospitalization and patients’ APT can be switched to ACT if paroxysmal atrial fibrillation (pAF) is detected later on. Thus, we may have overestimated early discontinuation rate of APT after ischemic stroke. However, warfarin was primarily used for ACT during the observation period of this study (new oral anticoagulants were not covered by Korean health insurance prior to 2015) and it is virtually unfeasible to accurately derive the adherence of warfarins because their dose and prescriptions need to be adjusted every time based on the patients’ time in therapeutic range. Also, it has been reported that the extent of diagnostic work-ups for detection of pAF differs greatly among institutions and follow-up electrocardiogram (ECG) is not performed after discharge in nearly 90% of cryptogenic strokes [50]. Moreover, among all cryptogenic strokes, only 1.4% are detected as pAF at 6 months and 2.0% at 12 months with routine ECG monitoring and the detection rates remains to be low even with an insertable cardiac monitoring (8.9% at 6 months and 12.4% at 12 months) [51]. In addition, premature discontinuation rate of APT for this study was mostly consistent with previous studies. Thus, we believe that the influence of this limitation of not assessing the ACT status of participants on overall results would be relatively low. Fourth, claim-based measurement of persistence with APT also has some limitations. Some participants may have obtained antiplatelets from a different source (e.g. sharing drugs with others, drug samples), and filling a prescription does not always mean that the drug was actually taken by patients [52, 53]. Nevertheless, measuring persistence using pharmacy refill and claims data is generally viewed objectively as it accurately reflects the real-world situations, owing to the large study populations of the database [54]. In addition, the use of over-the-counter (OTC) aspirin also could not be reviewed in this study since this variable is not included in the NHIS-NSC. However, we presume that most patients with ischemic stroke would take prescribed aspirin rather than OTC aspirin, since the former can be obtained at a discounted price via coverage by the Korean health insurance system [55]. Moreover, a previous simulation study confirmed that in many circumstances, the results based on prescription claims data are still valid despite missed OTC exposures [56]. Lastly, the present study did not specify the type of concomitant drugs taken by the patients. Even if the participants were prescribed with same number of medications, the effect on persistence may differ by type or component each concomitant drug. However, when analyzing with this kind of real-world data, it is very difficult to identify every single prescribed drug of each patients during the whole study period. Therefore, we decided to include the polypharmacy status of the patients by averaging total number of prescribed drugs during the observation period.

## Conclusions

In this retrospective cohort study, the early discontinuation of antiplatelets after ischemic stroke was commonly observed. The following factors independently predicted the premature discontinuation of antiplatelets: the type of initiated antiplatelets, age 70 years or above, lower income, rural residency, less than 4 total prescribed drugs, and a CCI score of 6 or higher. Therefore, by understanding factors influencing premature discontinuation, a more strategic approach is required for the physicians to improve persistence with antiplatelets among ischemic stroke survivors.

## Data Availability

Data are derived from the National Health Insurance service (NHIS). Interested researchers can request access to the data from the NHIS. The detailed information for data access of NHIS could be obtained from the NHIS website (www.nhis.or.kr).

## Abbreviations

aOR: Adjusted odds ratio
ACT: Anticoagulation therapy
APT: Antiplatelet therapy
ATC: Anatomical Therapeutic Chemical
CCI: Charlson comorbidity index
CI: Confidence interval
CT: Computed tomography
DAPT: Dual antiplatelet therapy
ECG: Electrocardiogram
GI: Gastrointestinal
ICD: International Classification of Disease
IRB: Institutional review board
MI: Myocardial infarction
MRI: Magnetic resonance imaging
NHIS: National Health Insurance Service
NHIS-NSC: National Health Insurance Service-National Sample Cohort
OR: Odds ratio
OTC: Over-the-counter
pAF: Paroxysmal atrial fibrillation
SD: Standard deviation
WHO: World Health Organization

## References

[1] Collaborators GLRoS (2018). Global, regional, and country-specific lifetime risks of stroke, 1990 and 2016. N Engl J Med.

[2] Johnson CO, Nguyen M, Roth GA, Nichols E, Alam T, Abate D, Abd-Allah F, Abdelalim A, Abraha HN, Abu-Rmeileh NM (2019). Global, regional, and national burden of stroke, 1990–2016: a systematic analysis for the global burden of disease study 2016. Lancet Neurol.

[3] Benjamin EJ, Muntner P, Alonso A, Bittencourt MS, Callaway CW, Carson AP, Chamberlain AM, Chang AR, Cheng S, Das SR, Delling FN, Djousse L, Elkind MSV, Ferguson JF, Fornage M, Jordan LC, Khan SS, Kissela BM, Knutson KL, Kwan TW, Lackland DT, Lewis TT, Lichtman JH, Longenecker CT, Loop MS, Lutsey PL, Martin SS, Matsushita K, Moran AE, Mussolino ME, O'Flaherty M, Pandey A, Perak AM, Rosamond WD, Roth GA, Sampson UKA, Satou GM, Schroeder EB, Shah SH, Spartano NL, Stokes A, Tirschwell DL, Tsao CW, Turakhia MP, VanWagner L, Wilkins JT, Wong SS, Virani SS, American Heart Association Council on Epidemiology and Prevention Statistics Committee and Stroke Statistics Subcommittee (2019). Heart disease and stroke Statistics-2019 update: a report from the American Heart Association. Circulation.

[4] van Wijk I, Kappelle LJ, van Gijn J, Koudstaal PJ, Franke CL, Vermeulen M, Gorter JW, Algra A, Li LACsg (2005). Long-term survival and vascular event risk after transient ischaemic attack or minor ischaemic stroke: a cohort study. Lancet.

[5] Touze E, Varenne O, Chatellier G, Peyrard S, Rothwell PM, Mas JL (2005). Risk of myocardial infarction and vascular death after transient ischemic attack and ischemic stroke: a systematic review and meta-analysis. Stroke.

[6] Trialists’Collaboration AJB (2002). Collaborative meta-analysis of randomised trials of antiplatelet therapy for prevention of death, myocardial infarction, and stroke in high risk patients. BMJ.

[7] Hackam DG, Spence JD (2019). Antiplatelet therapy in ischemic stroke and transient ischemic attack an overview of major trials and Meta-analyses. Stroke.

[8] Kernan WN, Ovbiagele B, Black HR, Bravata DM, Chimowitz MI, Ezekowitz MD, Fang MC, Fisher M, Furie KL, Heck DV, Johnston SC, Kasner SE, Kittner SJ, Mitchell PH, Rich MW, Richardson D, Schwamm LH, Wilson JA, American Heart Association Stroke Council, Council on Cardiovascular and Stroke Nursing, Council on Clinical Cardiology, and Council on Peripheral Vascular Disease (2014). Guidelines for the prevention of stroke in patients with stroke and transient ischemic attack: a guideline for healthcare professionals from the American Heart Association/American Stroke Association. Stroke.

[9] Kolandaivelu K, Leiden BB, O'Gara PT, Bhatt DL (2014). Non-adherence to cardiovascular medications. Eur Heart J.

[10] Bushnell C, Olson D, Zhao X, Pan W, Zimmer L, Goldstein L, Alberts M, Fagan S, Fonarow G, Johnston SC (2011). Secondary preventive medication persistence and adherence 1 year after stroke. Neurology.

[11] Glader E-L, Sjölander M, Eriksson M, Lundberg M (2010). Persistent use of secondary preventive drugs declines rapidly during the first 2 years after stroke. Stroke.

[12] Bushnell CD, Zimmer LO, Pan W, Olson DM, Zhao X, Meteleva T, Schwamm L, Ovbiagele B, Williams L, LaBresh KA (2010). Persistence with stroke prevention medications 3 months after hospitalization. Arch Neurol.

[13] De Schryver E, van Gijn J, Kappelle L, Koudstaal P, Algra A (2005). Non–adherence to aspirin or oral anticoagulants in secondary prevention after ischaemic stroke. J Neurol.

[14] Ji R, Liu G, Shen H, Wang Y, Li H, Peterson E, Wang Y (2013). Persistence of secondary prevention medications after acute ischemic stroke or transient ischemic attack in Chinese population: data from China National Stroke Registry. Neurol Res.

[15] Weimar C, Cotton D, Sha N, Sacco RL, Bath PM, Weber R, Diener HC, group Ps (2013). Discontinuation of antiplatelet study medication and risk of recurrent stroke and cardiovascular events: results from the PRoFESS study. Cerebrovasc Dis.

[16] Burke JP, Sander S, Shah H, Zarotsky V, Henk H (2010). Impact of persistence with antiplatelet therapy on recurrent ischemic stroke and predictors of nonpersistence among ischemic stroke survivors. Curr Med Res Opin.

[17] Ho PM, Bryson CL, Rumsfeld JS (2009). Medication adherence: its importance in cardiovascular outcomes. Circulation.

[18] Sabaté E, Sabaté E (2003). Adherence to long-term therapies: evidence for action.

[19] Lee J, Lee JS, Park S-H, Shin SA, Kim K (2016). Cohort profile: the national health insurance service–national sample cohort (NHIS-NSC), South Korea. Int J Epidemiol.

[20] Cheol Seong S, Kim Y-Y, Khang Y-H, Heon Park J, Kang H-J, Lee H, Do C-H, Song J-S, Hyon Bang J, Ha S (2017). Data resource profile: the national health information database of the National Health Insurance Service in South Korea. Int J Epidemiol.

[21] Nour M, Liebeskind DS (2011). Brain imaging in stroke: insight beyond diagnosis. Neurotherapeutics.

[22] Kim J, Bushnell CD, Lee HS, Han SW (2018). Effect of adherence to antihypertensive medication on the long-term outcome after hemorrhagic stroke in Korea. Hypertension.

[23] Kim J, Lee HS, Nam CM, Heo JH (2017). Effects of statin intensity and adherence on the long-term prognosis after acute ischemic stroke. Stroke.

[24] Powers WJ, Rabinstein AA, Ackerson T, Adeoye OM, Bambakidis NC, Becker K, Biller J, Brown M, Demaerschalk BM, Hoh B, Jauch EC, Kidwell CS, Leslie-Mazwi TM, Ovbiagele B, Scott PA, Sheth KN, Southerland AM, Summers DV, Tirschwell DL, American Heart Association Stroke Council (2018). 2018 guidelines for the early Management of Patients with Acute Ischemic Stroke: a guideline for healthcare professionals from the American Heart Association/American Stroke Association. Stroke.

[25] Organization WH (2006). The anatomical therapeutic chemical classification system with defined daily doses (ATC/DDD).

[26] Sundararajan V, Henderson T, Perry C, Muggivan A, Quan H, Ghali WA (2004). New ICD-10 version of the Charlson comorbidity index predicted in-hospital mortality. J Clin Epidemiol.

[27] Al AlShaikh S, Quinn T, Dunn W, Walters M, Dawson J (2016). Predictive factors of non-adherence to secondary preventative medication after stroke or transient ischaemic attack: a systematic review and meta-analyses. Eur Stroke J.

[28] Rosenstock IM, Strecher VJ, Becker MH (1988). Social learning theory and the health belief model. Health Educ Q.

[29] Rosenstock IM. Why people use health services. The Milbank Q. 2005;83(4). 10.1111/j.1468-0009.2005.00425.x.

[30] Kim D, Park J-M, Kang K, Cho Y-J, Hong K-S, Lee KB, Park TH, Lee SJ, Kim JG, Han M-K (2019). Dual versus mono antiplatelet therapy in large atherosclerotic stroke: a retrospective analysis of the Nationwide multicenter stroke registry. Stroke.

[31] Kim D, Lee S-H, Joon Kim B, Jung K-H, Yu K-H, Lee B-C, Roh J-K, investigators KSR (2013). Secondary prevention by stroke subtype: a nationwide follow-up study in 46 108 patients after acute ischaemic stroke. Eur Heart J.

[32] Lavie CJ, Howden CW, Scheiman J, Tursi J (2017). Upper gastrointestinal toxicity associated with long-term aspirin therapy: consequences and prevention. Curr Probl Cardiol.

[33] Poh CL, Chan M, Lau C, Teo SG, Low A, Tan HC, Lee CH (2011). Prevalence and predictors of premature discontinuation of dual antiplatelet therapy after drug-eluting stent implantation: importance of social factors in Asian patients. Intern Med J.

[34] Committee CS (1996). A randomised, blinded, trial of clopidogrel versus aspirin in patients at risk of ischaemic events (CAPRIE). Lancet.

[35] Grant RW, O’Leary KM, Weilburg JB, Singer DE, Meigs JB (2004). Impact of concurrent medication use on statin adherence and refill persistence. Arch Intern Med.

[36] Kim SJ, Kwon OD, Han EB, Lee CM, Oh S-W, Joh H-K, et al. Impact of number of medications and age on adherence to antihypertensive medications: A nationwide population-based study. Medicine. 2019;98(49). 10.1097/MD.0000000000017825. 10.1097/MD.0000000000017825 PMC6919523 31804305

[37] Kabboord AD, van Eijk M, Fiocco M, van Balen R, Achterberg WP (2016). Assessment of comorbidity burden and its association with functional rehabilitation outcome after stroke or hip fracture: a systematic review and meta-analysis. J Am Med Dir Assoc.

[38] Karatepe AG, Gunaydin R, Kaya T, Turkmen G (2008). Comorbidity in patients after stroke: impact on functional outcome. J Rehabil Med.

[39] Caballero PEJ, Espuela FL, Cuenca JCP, Moreno JMR, Zamorano JDP, Naranjo IC (2013). Charlson comorbidity index in ischemic stroke and intracerebral hemorrhage as predictor of mortality and functional outcome after 6 months. J Stroke Cerebrovasc Dis.

[40] Desrosiers J, Noreau L, Rochette A, Bourbonnais D, Bravo G, Bourget A (2006). Predictors of long-term participation after stroke. Disabil Rehabil.

[41] Holmes HM, Luo R, Hanlon JT, Elting LS, Suarez-Almazor M, Goodwin JS (2012). Ethnic disparities in adherence to antihypertensive medications of medicare part D beneficiaries. J Am Geriatr Soc.

[42] Cho MH, Shin DW, Chang S-A, Lee JE, Jeong S-M, Kim SH, Yun JM, Son K (2018). Association between cognitive impairment and poor antihypertensive medication adherence in elderly hypertensive patients without dementia. Sci Rep.

[43] Salas M, In't Veld BA, van der Linden PD, Hofman A, Breteler M, Stricker BH (2001). Impaired cognitive function and compliance with antihypertensive drugs in elderly: the Rotterdam study. Clin Pharmacol Ther.

[44] Turner A, Hochschild A, Burnett J, Zulfiqar A, Dyer CB (2012). High prevalence of medication non-adherence in a sample of community-dwelling older adults with adult protective services-validated self-neglect. Drugs Aging.

[45] Quadros AS, Welter DI, Camozzatto FO, Chaves Á, Mehta RH, Gottschall CA, Lopes RD (2011). Identifying patients at risk for premature discontinuation of thienopyridine after coronary stent implantation. Am J Cardiol.

[46] Spertus J, Kettelkamp R, Vance C, Decker C (2006). Prevalence, predictors and outcomes of premature discontinuation of thienopyridine therapy after drug-eluting stent placement. Circulation.

[47] Murphy GK, McAlister FA, Weir DL, Tjosvold L, Eurich DT (2014). Cardiovascular medication utilization and adherence among adults living in rural and urban areas: a systematic review and meta-analysis. BMC Public Health.

[48] Goins RT, Williams KA, Carter MW, Spencer SM, Solovieva T (2005). Perceived barriers to health care access among rural older adults: a qualitative study. J Rural Health.

[49] Park J-H, Shin Y, Lee S-Y, Lee SI (2008). Antihypertensive drug medication adherence and its affecting factors in South Korea. Int J Cardiol.

[50] Rizos T, Quilitzsch A, Busse O, Haeusler KG, Endres M, Heuschmann P, Veltkamp R (2015). Diagnostic work-up for detection of paroxysmal atrial fibrillation after acute ischemic stroke: cross-sectional survey on German stroke units. Stroke.

[51] Sanna T, Diener H-C, Passman RS, Di Lazzaro V, Bernstein RA, Morillo CA, Rymer MM, Thijs V, Rogers T, Beckers F (2014). Cryptogenic stroke and underlying atrial fibrillation. N Engl J Med.

[52] Pladevall M, Williams LK, Potts LA, Divine G, Xi H, Lafata JE (2004). Clinical outcomes and adherence to medications measured by claims data in patients with diabetes. Diabetes Care.

[53] Osterberg L, Blaschke T (2005). Adherence to medication. N Engl J Med.

[54] Lehmann A, Aslani P, Ahmed R, Celio J, Gauchet A, Bedouch P, Bugnon O, Allenet B, Schneider MP (2014). Assessing medication adherence: options to consider. Int J Clin Pharm.

[55] Kim Y-J, Choi N-K, Kim M-S, Lee J, Chang Y, Seong J-M, Jung S-Y, Shin J-Y, Park J-E, Park B-J (2015). Evaluation of low-dose aspirin for primary prevention of ischemic stroke among patients with diabetes: a retrospective cohort study. Diabetol Metabolic Syndrome.

[56] Yood MU, Campbell UB, Rothman KJ, Jick SS, Lang J, Wells KE, Jick H, Johnson CC (2007). Using prescription claims data for drugs available over-the-counter (OTC). Pharmacoepidemiol Drug Saf.

